# Fruit and vegetable discards preserved with sodium metabisulfite as a high-moisture ingredient in total mixed ration for ruminants: effect on *in vitro* ruminal fermentation and *in vivo* metabolism

**DOI:** 10.5713/ajas.19.0596

**Published:** 2019-10-22

**Authors:** Farhad Ahmadi, Won Hee Lee, Young-Kyoon Oh, Keunkyu Park, Wan Sup Kwak

**Affiliations:** 1 College of Medical Life Sciences and College of Sanghur Life Science, Konkuk University, Chungju 27478, Korea; 2 Animal Nutrition and Physiology Team, National Institute of Animal Science, Rural Development Administration, Wanju 55365, Korea

**Keywords:** Fruit and Vegetable Waste, Nutrient Digestibility, Ruminant, Sodium Metabisulfite

## Abstract

**Objective:**

Our recent series of laboratory- and large-scale experiments confirmed that under aerobic and anaerobic conditions, sodium metabisulfite (SMB) was effective in preserving nutrients and antioxidant capacity of highly perishable fruit and vegetable discards (FVD). Hence, the purpose of this study was to examine how partial inclusion of SMB-treated FVD in total mixed ration (TMR) influences *in vitro* ruminal fermentation, whole-tract digestibility, nitrogen metabolism, blood metabolites, and voluntary feed intake of sheep.

**Methods:**

The FVD were mixed thoroughly with 6 g SMB/kg wet biomass and kept outdoors under aerobic conditions for 7 days. Four TMRs including four levels of SMB-treated FVD (as-fed basis) at 0%, 10%, 20%, and 30% (equaling to 0%, 1.9%, 3.8%, and 5.7% on dry matter basis, respectively), were prepared as replacement for corn grain. The ruminal fermentation metabolites were studied using an *in vitro* gas production test. Four mature male Corriedale sheep were assigned at random to the 4 diets for two separate sub-experiments; i) digestibility trial with four 21-d periods, and ii) voluntary feed intake trial with four 28-d periods.

**Results:**

Inclusion of SMB-treated FVD in the TMR tended to quadratically increase partitioning factor. No effect was seen on total-tract digestibility of organic matter, ether extract, crude protein, and acid detergent fiber, except for neutral detergent fiber digestibility that tended to linearly increase with increasing SMB-treated FVD in the TMR. The progressive increase of FVD preserved with SMB in the diet had no effect on nitrogen metabolism. Treatment had no effect on serum antioxidant capacity and blood metabolites assayed. Voluntary feed intake was not impaired by inclusion of SMB-treated FVD in the TMR.

**Conclusion:**

It appears that FVD preserved with SMB can be safely incorporated into TMR as replacement of corn grain without impairment of nutrient metabolism and feed intake.

## INTRODUCTION

The growing trend in the demand for more animal protein by human populations as well as reduced harvests owing to climate change and diminished land availability imply an intensified demand for livestock feed in the foreseeable future [1]. In animal production systems, feed constitutes the majority of input costs [2]. Hence, the development of animal feed from human-inedible resources offers a promising route to potentially controlling feed costs, especially in countries where feed supply is limited. In addition, owing to their inherent efficiency to transform the human-inedible inputs into meat and milk, the use of human-inedible resources in animal diet contributes to a net gain in food supply [3].

Globally, a huge portion of fruit and vegetable is discarded. Underutilization of fruit and vegetable discards (FVD) in some parts of the world results in their decomposition in landfills, which causes environmental concerns [4]. According to estimates of the United Nations Food and Agriculture Organization, about 45% to 55% of all fruit and vegetable produced worldwide are lost and wasted at different stages of the food-supply chain [4]. The use of FVD as animal feed opens a potential route to acquiring more of the added value of FVD which contributes to environmental protection, sustainable development of animal production, and efficient utilization of this waste resource.

Although a number of studies attempted to use FVD as a feed ingredient in animal diet [5], FVD is a relatively new class of feed ingredient potentially rich in vitamins, minerals, and phytonutrients which may provide additional benefits for animals beyond a simple feed ingredient. According to the experimental data obtained over two years in our laboratory [6,7], FVD is high in moisture (85% to 88%) and soluble sugars (44% to 63%; dry matter [DM] basis), which speed up the microbial deterioration and therefore causes environmental problems [6,7]. Mixing FVD with dry feeds to achieve appropriate moisture content, facilitates the storage and handling, minimizes the effluent loss, and decreases the costs related to drying and transportation of high-moisture feedstocks [8]. The use of high-moisture FVD in total mixed ration (TMR) also contributes to the moisture requirement for proper microbial fermentation.

However, our last experiment found the energetic cost with respect to DM and nutrient loss was substantial when FVD was stored without preservatives and with or without moisture absorbents (corn gluten feed+almond hulls) under either aerobic or anaerobic conditions [7]. Previously, our research team developed successful preservation methods using sodium metabisulfite (SMB; Na_2_S_2_O_5_) that confirmed the efficacy of this sulfur-based preservative in delaying the quality deterioration and retaining the nutrients and antioxidant constituents of FVD under both aerobic and anaerobic conditions [7,8]. The use of SMB could also be beneficial as it can contribute to a part of the sulfur requirement of the animal.

A positive relationship exists between the dietary water-soluble carbohydrates (WSC) content and the efficiency of N utilization in ruminants [9]. Based on this evidence, our initial hypothesis was that the increased supply of WSC through the dietary inclusion of FVD as a rich source of WSC would improve the N-use efficiency. Our second hypothesis was that FVD as a high-energy source (high content of non-fibrous carbohydrates) could partially replace corn grain (a conventional energy source) in ruminant diets. Thus, this investigation aimed to understand how the partial inclusion of FVD preserved with SMB in TMR affects ruminal fermentation metabolites, whole-tract nutrient digestibility, N metabolism, blood metabolites, and voluntary feed intake of sheep as a model ruminant species.

## MATERIALS AND METHODS

The Institutional Animal Care and Use Committee of the Konkuk University reviewed and approved the procedures involving animals. Before the initiation of the *in vivo* metabolism study, the sheep were treated against endoparasites and ectoparasites. Sheep were also allowed to habituate to the environmental and experimental conditions for 3 weeks before the commencement of experiments.

### Preparation of fruit and vegetable discards

The quantity of major individual ingredients discarded during the month (June 2018) constituting more than 90% of total discards were surveyed from the input and output data at a commercial large-scaled packing house (E-mart Fresh Center, Icheon, Korea). Approximately, 700 kg of the discards (22.4% onion, 20.6% lemon, 17.2% potato, 16.1% tomato, 14.4% plum, 5.3% sweat potato, 2.2% green onion stalk, and 1.8% paprika) were collected from the packing house during two consecutive days. The discards were roughly sliced into 3 to 4 cm pieces and thoroughly mixed with 6 g SMB/kg fresh FVD mass using a horizontal feed mixer (DDK-801 M, Daedong Tech. Co., Gyeongsan, Korea). The blends were transferred to buckets (120 L capacity) and kept outdoors (shaded area) under aerobic exposure for seven days. The 7-day aerobic preservation was selected based on our previous on-site survey in the packing house where FVD is accumulated on-site and transported to the recycling center typically within a week [6]. The FVD after aerobic exposure were minced using a meat grinder to minimize differences in particle size and to provide homogenous mixing with TMR ingredients. The minced FVD was then divided into the monthly portions (about 80 kg) in sealed plastic bags and stored at −20°C, until being thawed and mixed with the diet ingredients. Nutrient composition profile of FVD before and after the aerobic challenge is presented in Table 1.

### Preparation of experimental diets

Four TMRs included four levels of SMB-treated FVD in TMR (on a DM basis) as: 0%, 1.9%, 3.8%, and 5.7% (equaling to 0%, 10%, 20%, and 30% on as-fed basis, respectively), replacing corn grain in the diet equaling 0% to 33%. The target DM content of TMRs was adjusted with water to about 62%, which is within the normal DM range of TMR. Owing to the high moisture content of FVD (DM = 12.9%), the highest level of FVD inclusion in TMR was decided as 30% on an as-fed basis, equaling to 5.7% on a DM basis. The feed ingredients of the basal diet (DM basis) were 40.0% timothy hay, 35.0% corn gluten feed, 17.1% corn grain, and 6.0% almond hulls. Experimental diets were formulated to provide similar levels of energy (total digestible nutrient [TDN] = 67.8% of DM) and crude protein (CP; 14.2% of DM), to meet the nutrient requirements of sheep as recommended by the National Research Council [10]. The TDN content of FVD was estimated to be 80.2%, which was calculated using the equation of Conrad et al [11]. Timothy hay was chopped to 2-3 cm lengths with a forage cutter (Model: SC-1000; Hwang So Agri-Machinery Co., Ltd, Daegu, Korea). The diet ingredients were mixed in a DDK-801 feed mixer (Daedong Tech. Co., Korea) for 15 min. The chemical composition of individual ingredients used in the formulation of TMR is reported in Table 2. The ingredient and chemical composition of experimental TMRs is presented in Table 3.

### *In vitro* incubation experiment

To investigate the effect of SMB-treated FVD on ruminal fermentation patterns, before the *in vivo* sheep metabolism trial, the experimental diets (*i.e.*, diets with 0%, 10%, 20%, and 30% FVD) were subjected to the *in vitro* gas production test. Two hours after the morning feeding, rumen fluid was harvested from two ruminally-cannulated Hanwoo steers (body weight [BW] = 555±16.4 kg; mean±standard deviation [SD]) and immediately transferred in thermos flasks to the laboratory. The steers were fed a TMR (32.2% tall fescue hay, 23.1% cracked corn grain, 20.3% pineapple by-product, 16.5% rice straw, 7.3% soybean meal, 0.25% salt, 0.25% limestone, and 0.10% vitamin-mineral premix), twice daily, at 0900 and 1800 h. The ruminal fluids were composited, filtered through a 1-mm sieve, and mixed at a ratio of 1:4 (v/v) with the pre-warmed buffer [12]. The buffered rumen fluid was bubbled with continuous flushing of pure warm CO_2_. In sub-Exp. 1, to each volume-calibrated 100-mL glass syringe 200±5 mg of dried sample (10 replicates per sample) and 30 mL of the buffered rumen fluid were added [12]. The syringes were incubated at 39°C in a shaking incubator (JSSI-100T, JS Research Inc., Gongju, Korea). Incubations were terminated with half of the syringes (five per treatment) after 24 h, to determine the ruminal pH (HI 9321, Hanna Instruments, Woonsocket, RI, USA) and rumen fermentation metabolites (NH_3_-N and total lactate). If gas volumes in any of the syringes reached above 85 mL, the gas volume was recorded, and the plunger pushed back to the original volume of 30 mL. The remaining syringes continued to incubate until 96 h (totally 15 time-point readings). The incubation time when GP reached half of asymptotic gas production, termed GP*t*_1/2_, was estimated according to the equation of Blümmel et al [13]. For NH_3_-N analysis, a portion of supernatant (1.5 mL) was mixed with 0.3 mL of sulfuric acid (1%; v/v). NH_3_-N was determined calorimetrically [14]. Total lactate was quantified using a colorimetric method [15].

The sub-Exp. 2 followed the protocol suggested by Blümmel et al [13] to estimate the partitioning factor (PF), which is an index of the efficiency of microbial biomass synthesis. The ruminal fluid preparation and incubation conditions were similar to those explained in sub-Exp. 1. Briefly, to each glass syringe 500±10 mg of dried sample (5 replicates per each sample) and 40 mL of the buffered rumen fluid were added. Incubations were terminated when GP reached half of asymptotic gas production. The entire content of each syringe was then transferred into a conical tube and centrifuged for 30 min for 20,000 *g* at 4°C. Efficiency of microbial biomass synthesis was estimated as the proportion of organic matter (OM) degraded to GP*t*_1/2_, termed PF. After centrifugation of the content of each syringe and removal of the supernatant, the pellet was carefully recovered and refluxed with neutral detergent solution (100 mL; without sodium sulfite and amylase) for 1 h. This enabled the separation of the microbial mass from the undegraded substrate. The content was then filtered through filter crucibles and washed several times with boiling distilled water until the remaining neutral detergent was removed. The residual solid recovered was dried (100°C for 24 h), weighed, and ashed (500°C for 3 h) for OM determination. True OM degradability was calculated as the difference between OM substrate incubated (mg) and residual OM (mg). In both sub-experiments, five syringes with only buffered rumen fluid were incubated to serve as blank. The amount of gas produced in blank syringes was averaged and subtracted from the GP of experimental samples.

### *In vivo* digestibility experiment

Four mature male Corriedale sheep (BW = 50.2±3.82 kg, mean ±SD) were housed in individual metabolism crates (0.80× 1.40 m), and allotted at random to 1 of 4 TMRs in a 4×4 Latin square design. Treatment periods were 21 d in length each (14-d adaptation). The estimated portions of TMR were made monthly, divided into the daily allotments in sealed nylon bags (about 3 kg/bag), and stored at −20°C. About 12 h before the morning feeding, the daily portions were thawed at room temperature prior to being offered to the sheep. Sheep received their respective diets (DM basis) at 2% of their BW daily in equal allotments twice daily (0900 and 1800 h). Throughout the experiment, sheep had free access to fresh water at all times, which was renewed at each feeding.

Total feces excreted daily was collected for each sheep, mixed thoroughly, and a representative sample (about 20% w/w) was stored at −20°C for later analysis. After the sample collection was completed, the fecal samples were thawed overnight, pooled by sheep within each period, and then dried at 55°C for 4 d for DM content determination. Buckets containing 100 mL of 6 *N* HCl were used for daily urine collection [16]. After urine volume measurement, an aliquot of urine (10% of total urine) was collected and stored at −20°C. The urine samples were pooled by sheep within each period and prepared for later analysis. N balance was calculated by subtraction of average daily N intake from the average daily N excreted in the feces and urine. From the difference in the amount of nutrient intake and that excreted in feces, total-tract digestibility coefficients were estimated.

### Voluntary feed intake experiment

After the digestibility trial, the same sheep were used in a voluntary feed intake experiment. Similar to the digestion trial, sheep were distributed randomly to 1 of 4 TMRs (Table 3) in a 4×4 Latin square design (28-d periods; 21-d adaptation). The diets were offered in equal allotments twice daily (0900 and 1800 h) in amounts adequate to allow voluntary access (about 5% orts). Before the morning feeding, orts were weighed daily to calculate dry matter intake (DMI). The DMI was also expressed as a unit of metabolic BW, which was calculated using the average value of initial and final BW for each period raised to the power of 0.75. Fresh water was renewed at each feeding. Water intake was measured twice daily from d 22 to 28, considering the water evaporation loss during the 24 h. On two consecutive days (d 27 and 28), about 4 h after the morning feeding, blood samples were collected by jugular venipuncture into evacuated tubes using a 22-gauge needle. Harvested blood was divided into two portions, without (BD Vacutainer SST II Advance; Plymouth, UK) or with anticoagulant (BD Vacutainer, K2 EDTA, Franklin Lakes, NJ, USA). Serum was harvested by centrifugation (1,200 *g* for 20 min) and stored at −20°C for later analysis.

### Laboratory analyses

An extract of FVD was obtained by mixing a 20-g FVD sample with 200 mL of sterilized distilled water for 10 min. The suspension was filtered through two layers of cheesecloth, and silage pH was immediately measured with an electrode (HI 9321, Hanna Instruments, USA). The filtrate was centrifuged at 4°C for 20 min at 10,000 *g* and analyzed for lactic acid [15], WSCs [17], and NH_3_-N [14]. Ethanol and acetic acid were quantified using the commercial kits according to the supplier’s protocol (Megazyme International Ireland Ltd., Wicklow, Ireland). Total numbers of bacteria and lactic acid bacteria were counted using the spread-plating methods. Yeast and mold were visually differentiated on yeast extract glucose chloramphenicol agar (Difco Laboratories Inc., Detroit, MI, USA), after incubation at 25°C±1°C for 5 d. A representative sample of FVD, before and after aerobic exposure with SMB, was obtained and sent to a commercial laboratory to check for total aflatoxin presence using a high-performance liquid chromatography method.

A methanolic extraction of freeze-dried FVD was obtained to determine total phenolics and antioxidant capacity of FVD. The homogenized ground FVD (1 g) was mixed with 10 mL of 80% methanol and shaken using a rotary shaker (250 rpm for 1 h at 30°C). The suspension was centrifuged (4,000 *g* for 10 min at 4°C). After the harvest of supernatant, the residual solids were re-extracted three more times with the same procedure. The total phenolic content of the supernatant was quantified using the Folin–Ciocalteu reagent, as previously described in detail [6]. For quantifying the 2, 2-diphenyl-1-picrylhydrazyl (DPPH) free radical-scavenging activity, a portion of the supernatant (100 μL) was reacted at room temperature with 3.9 mL of the methanolic solution of 60 μM DPPH^·^. After 30-min incubation, absorbance was read at 515 nm.

Urine was analyzed for N using the Kjeldahl method [18]. The procedures outlined by the Association of Official Analytical Chemists [18] were used for determination of DM, ether extract, CP, crude ash, ash-corrected neutral detergent fiber (NDF) and acid detergent fiber (ADF) contents of feed and fecal samples. Lignin content of FVD was quantified after solubilization of cellulose with 72% H_2_SO_4_. The diet pH was determined with a pH meter (HI 9321, Hanna Instruments, USA) after mixing 30 g diet sample with 120 mL distilled water and stirring for 30 s [16]. The representative samples of TMR, dried in a freeze drier, were digested with perchloric acid and their mineral composition was determined using an ICP-OES (5300DV, Perkin Elmer, Billerica, MA, USA), according to the Association of Official Analytical Chemists protocol [18].

### Blood metabolites

The harvested bloods in vacutainer tubes were sent to a commercial laboratory (Green Cross Corporation, Yongin, Korea) for the analysis of serum metabolites using an Automatic Biochemical Analyzer (Hitachi 7170A, Hitachi Ltd., Tokyo, Japan). Phosphorus, potassium, sodium, and chlorine levels were quantified using an ICP-OES (5300DV, Perkin Elmer, USA). The original method of Erel [19] was used to determine total antioxidant capacity of serum samples. For 2,2′-azinobis (3- ethylbenzothiazoline-6-sulfonic acid) radical cation (ABTS^.+^) generation, a 7-mM ABTS solution was reacted with potassium persulfate (2.45 mM). The bleaching of ABTS^.+^ by antioxidants present in the serum sample was monitored spectrophotometrically (at 734 nm). Trolox was used as the standard (0 to 2 mM), and the results were expressed in μmol Trolox equivalent/mL.

### Statistical analysis

The difference between treatments in Table 1 was identified using a *t*-test using the PROC TTEST of SAS [20]. The *in vitro* data were analyzed in a completely randomized design using the PROC MIXED of SAS. The *in vivo* data were analyzed as a 4×4 Latin square using the PROC MIXED of SAS. The model below was used for analysis:

$${\text{Y}}_{\text{ijk}}=\mu +{\text{S}}_{\text{i}}+{\text{P}}_{\text{j}}+{\text{T}}_{\text{k}}+{ɛ}_{\text{ijk}}$$

where, Y, μ, A, P, T, and ɛ denote the independent variable, mean, sheep, period, treatment, and random residual error, respectively. Individual sheep served as the experimental unit and treatments as the main factor. The treatment means were compared using the Tukey’s test. Orthogonal contrasts were constructed to evaluate the linear and quadratic effects using the CONTRAST statement of SAS. Significance level was noted at a p-value of <0.05.

## RESULTS

### Aerobic preservation of fruit and vegetable discards

Nutrient composition, metabolites, microbial population, and antioxidant capacity of FVD preserved with SMB before and after 7 d of aerobic exposure are presented in Table 1. In general, negligible differences were noted in the parameters analyzed, with the exception of NH_3_-N and acetic acid that tended to slightly increase (p = 0.08) and increased (p< 0.01) after aerobic exposure, respectively. No aflatoxin was detected in FVD before and after storage. Negligible change was observed in total phenolics and antioxidant capacity of FVD preserved with SMB.

### *In vitro* fermentation experiment

Gas production and ruminal fermentation metabolites of TMRs with different levels of SMB-treated FVD, are presented in Table 4. Despite the linear reduction (p<0.01) of GP*t*_1/2_ with increasing the FVD levels as replacement to corn grain, true OM degradability was not affected with treatment, resulting in a quadratic increasing trend (p = 0.09) in PF value. Treatment had no effect on ruminal pH and NH_3_-N (after 24-h fermentation).

### *In vivo* sheep metabolism experiment

In general, throughout both digestibility and voluntary feed intake trials, none of the sheep showed any apparent illness over the course of experiment. Apparent nutrient digestibility coefficients, N metabolism, and voluntary feed intake of sheep fed TMR with different levels of FVD are presented in Table 5. The effect of period or sheep was not significant for any parameter analyzed, which was expected as the environmental conditions were controlled and remained stable throughout the trial. No orts remained for any diets during the digestibility experiment. The progressive increase of the FVD inclusion in the ration had no effect on apparent total-tract digestibility of OM, ether extract, CP, and ADF. However, NDF digestibility tended to increase linearly (p = 0.06) with the elevated levels of SMB-treated FVD in the diet. Treatment had no effect on N metabolism. The inclusion of SMB-treated FVD in TMR did not impair voluntary feed intake (kg DM/d or g/kg BW^0.75^/d). No effect was seen on voluntary water intake (kg/kg DMI) with increasing SMB-treated FVD in TMR. Treatment had no effect on fecal consistency. As presented in Table 6, none of the blood metabolites were affected with SMB-treated FVD inclusion in the diet.

## DISCUSSION

### Preservation of fruit and vegetable discards with sodium metabisulfite

Previously we observed that FVD started to putrefy promptly as they are accumulated outdoors, which is related to the high sugar and moisture content of FVD. Our preliminary experiments also found that the immediate ensiling of FVD was not an effective strategy in controlling microbial spoilage and nutrient loss, which was related to the high initial numbers of molds and yeasts existing on FVD biomass [7]. This necessitated the immediate use of a safe, effective, and cheap preservative suitable for animal feed applications. To meet these criteria, we selected SMB that showed successful preservative potential during the aerobic and anaerobic storage of FVD [6,7]. The extensive small- and pilot-scale investigations in our laboratory confirmed that 6 g SMB/kg wet FVD was the minimum amount that was quite effective in preserving the nutrients and antioxidant capacity of FVD biomass within a week [6,7]. The negligible changes in antioxidant activity of FVD preserved with SMB agrees with our previous results that suggested the protective function of SMB on the antioxidant capacity of FVD under aerobic and anaerobic storage [6].

### *In vitro* fermentation experiment

Principally, PF has been suggested as an index to express the efficiency of microbial biomass synthesis [13]. In fact, the rate of microbial growth in the rumen is largely determined by the ruminal availability of carbohydrates [21]. Lee et al [9] suggested that with increasing WSC inclusion, a microbial population shift occurred which favored the microbial protein synthesis in the rumen. According to the Cornell Net Carbohydrate and Protein System, the ruminal organisms capable of fermenting starches in high-moisture corn could contribute less in microbial protein production (about 18%) than the organisms that ferment the soluble sugars [22].

Contrary to our observation that the increased concentra tion of WSC through the elevated levels of FVD did not affect ruminal pH and ammonia *in vitro*, Lee et al [9] reported that with increasing WSC content of basal grass diet (1 to 1.75 times) through sugar infusion, linear reductions were seen in pH and NH_3_-N in an *in vitro* RUSITEC system. However, consistent with our results Penner and Oba [23] did not find any detectable difference in rumen ammonia concentration with increasing sugar concentration of the diet.

Generally, a decline in rumen pH is expected with feeding rapidly fermentable sugars relative to other carbohydrate fractions. However, in a review of literature, Oba [24] suggested feeding high-sugar diets did not necessarily decrease rumen pH. This was substantiated by the speculation that the greater supply of dietary sugar may contribute to the increased production of microbial mass, which in turn may decrease the availability of OM for fermentation acid production [24]. The conversion of sugars into microbial glycogen is another suggested theory, which may temporarily decrease the fermentation acid production in the rumen, thereby preventing decline in rumen pH [24].

### *In vivo* sheep metabolism experiment

One of the main focuses of the *in vivo* trial was to examine if the incremental levels of SMB in the diet would affect DMI and health of sheep. Previously, Nikolaev and Dzhidzheva [25] reported that 2.25 g SMB/kg of BW in sheep was the minimum lethal dose which resulted in feed refusal and severe health disorders. In the present study however, the level of SMB added to the diet (DM basis) was much lower than the minimum tolerable dose reported, which may explain why DMI and health of sheep remained unaffected with the different levels of SMB in the diet. Bird [26] suggested the intake of no more than 4 g sulfur per day in sheep should fulfill microbial and tissue sulfur requirements without adversely affecting DMI.

Consistent with our observation that the elevated levels of SMB-treated FVD had no effect on apparent digestibility of majority of the nutrients, previous studies with dairy cows [23] also did not find such effects when sugar was fed as replacement to corn grain at 4.4% to 4.7% of dietary DM. The observation that the inclusion of the FVD in TMR had no adverse effect on the apparent digestibility of fiber fraction indicates the ruminal pH was not affected with treatment, supporting the data of the *in vitro* experiment which showed the progressive increase of the SMB-treated FVD had no detectable effect on ruminal pH. Generally, the activity of ruminal microorganisms responsible for degradation of cellulose and hemicellulose is known to be inhibited when ruminal pH reduction occurs [21]. In support of our findings that the NDF digestibility tended to increase linearly with the elevated FVD inclusion level, Broderick and Radloff [22] reported that when the dietary sugar concentration was 7.2%, total-tract NDF digestibility was greatest. Our analysis of TMR composites collected during the experiment showed dietary NDF increased by 1.3% over the range of these diets (Table 3). This was caused by the progressive replacement of corn grain (with less NDF; 15.1%) with FVD (more NDF; 22.1%). A trend to linear increase in NDF digestibility with higher FVD level possibly happened because the elevated levels of SMB-treated FVD in the diet resulted in the greater intake of more digestible fiber.

Contrary to our finding that the elevated levels of SMB- treated FVD had negligible effect on DMI, Morrow et al [16] reported a reduction in DMI when the dietary S increased with Na_2_SO_4_ addition to the diet of lambs. In the current study, we used mature sheep in the voluntary feed intake trial, which may be less sensitive to the increased S content of the diet through SMB addition. Further experiments are needed to evaluate the dietary inclusion levels of SMB-treated FVD on growth performance of growing lambs. The lack of treatment effect on voluntary water intake agrees with the findings of Morrow et al [16] who reported the increase in dietary sodium by 26 to 38 g/d through Na_2_SO_4_ addition had no effect on water intake of lambs.

Although the data of *in vitro* study partially confirmed the hypothesis that the increased sugar concentration of the diet benefited the N-use efficiency (increased PF unit), this effect was not confirmed in the *in vivo* sheep metabolism study, as N metabolism and plasma urea N were not different across treatments. Many studies have criticized to extrapolate *in vitro* data to *in vivo* conditions, which highlights the careful interpretation of *in vitro* data [10]. In his review report, Oba [24] concluded the partial inclusion of sugars in ruminant diet does not necessarily improve the efficiency of N utilization. In contrast, studies with sheep suggested feeding sugars is associated with the decreased urinary N excretion and increased N retention [27]. The urinary and fecal excretion rate of N is directly associated with the amount of N ingested. This possibly occurred in this experiment as N intake was similar among treatments. As no orts remained in the digestibility trial for any of the diets offered, the similar N intake among sheep was expected as the diets were formulated to provide similar concentration of N (Table 3). Morrow et al [16] reported the addition of sodium sulfate (Na_2_SO_4_) to the diet (0.88%) had no effect on N excretion and retention, which was explained by the similar N intake among sheep.

### Blood metabolites

Adding S sources to the diet is reported to reduce plasma Cu concentration, and subsequently ceruloplasmin, a protein that follows plasma Cu concentrations [28]. The ruminal degradation of S compounds to sulfide is known to contribute to formation of insoluble copper sulfide, which therefore reduces the availability of dietary Cu [28]. Presently, the incremental levels of SMB-treated FVD led to the slight increase in the dietary S content; however, this increase had no effect on plasma ceruloplasmin. This observation might be related to the small increase in S content of diet (DM basis) which did not affect the S metabolism of sheep.

Contrary to our initial hypothesis that the antioxidant ca pacity of the FVD may contribute to the increased plasma antioxidant status of sheep, we found the incremental levels of SMB-treated FVD with potential antioxidant capacity had no effect on plasma antioxidant capacity of sheep. Similarly, previous studies adding the feed supplements with high antioxidant activity in ruminant diet failed to detect any effect in plasma antioxidant status. The complex regulation of endogenous antioxidant expression as well as the strong buffering capacity of the redox system in ruminants have been suggested to explain the lack of such effects [29].

## CONCLUSION

The partial inclusion of discarded fruits and vegetables preserved with metabisulfite as a high-moisture ingredient in replacement to corn grain in TMR did not impair nutrient metabolism and voluntary feed intake, and blood metabolites of sheep. The use of FVDs in ruminant diet may contribute to lessening the problem of solid waste disposal and presents an opportunity for decreasing the cost of animal diets especially in places where the provision of animal feed is prohibitive owing to high prices.

## Figures and Tables

**Table 1:** Nutrient composition and microbiological profile of fruit and vegetable discards preserved with sodium metabisulfite after 7 days under aerobic conditions

Items	Aerobic preservation, day		SEM	p-value

	0	7		
DM (%)	13.4	12.9	0.31	0.37
Crude protein (% of DM)	7.84	7.78	0.381	0.36
NDF (% of DM)	21.8	22.1	2.02	0.71
ADF (% of DM)	18.1	18.3	1.33	0.52
Ether extract (% of DM)	2.69	2.89	0.532	0.78
Ash (% of DM)	8.15	8.29	0.661	0.33
NFC (% of DM)	59.5	58.9	3.28	0.49
WSC (% of DM)	42.9	40.4	2.11	0.09
NH_3_-N (μg/g DM)	1.19	2.98	0.433	0.07
pH	3.76	3.79	0.042	0.44
Ethanol (mg/g DM)	0.98	1.22	0.221	0.18
Acetic acid (mg/g DM)	0.08	0.14	0.012	<0.01
Lactic acid (g/100 g DM)	0.04	0.02	0.013	0.42
Microbial population (log cfu/g wet biomass)				
Total bacteria	5.89	4.19	0.811	0.04
Lactic acid bacteria	4.54	<2.8	-	<0.01
Yeasts	4.69	2.95	0.642	<0.01
Molds	3.53	<2.8	-	<0.01
Total phenolics (mg gallic acid equivalent/g DM)	10.1	9.61	0.39	0.13
DPPH (mg ascorbic acid equivalent/g DM)	11.3	10.8	0.34	0.10
Total aflatoxin (ppb)	ND	ND	-	-

SEM, standard error of the mean; DM, dry matter; NDF, neutral detergent fiber; ADF, acid detergent fiber; NFC, non-fibrous carbohydrates; WSC, water-soluble carbohydrates; DPPH, 2, 2-diphenyl-1-picrylhydrazyl; ND, not detected.

**Table 2:** Nutrient composition of timothy hay, corn gluten feed, corn grain, and almond hulls (values based on % of DM; mean±standard deviation)

Feed ingredient	DM (%)	Crude protein	Ether extract	NDF	ADF	Ash	NFC
Timothy hay	90.2±0.06	10.8±0.81	1.11±0.02	73.2±2.18	36.1±1.33	8.19±0.52	6.70±0.77
Corn gluten feed	91.8±0.09	23.8±0.77	1.81±0.03	38.5±1.87	14.2±0.94	6.45±0.66	31.1±2.44
Corn grain, cracked	90.1±0.04	8.45±0.43	2.92±0.05	15.1±0.88	6.28±0.34	2.66±0.09	71.0±3.55
Almond hulls	93.2±0.11	4.88±0.09	0.83±0.06	70.9±3.33	49.2±2.77	10.1±0.67	13.2±1.22

DM, dry matter; NDF, neutral detergent fiber; ADF, acid detergent fiber; NFC, non-fibrous carbohydrates.

**Table 3:** Ingredients and nutrient composition of experimental rations (DM basis; n = 4)

Items	Level of FVD in TMR (% of DM)			

	0	1.9	3.8	5.7
Ingredients				
Timothy hay, chopped	40.0	40.0	40.0	40.0
Corn gluten feed	35.0	35.0	35.0	35.0
Corn grain, cracked	17.1	15.2	13.3	11.4
Almond hulls	6.0	6.0	6.0	6.0
FVD	0	1.9	3.8	5.7
Vitamin-mineral supplement1)	1.0	1.0	1.0	1.0
Limestone	0.6	0.6	0.6	0.6
Salt	0.3	0.3	0.3	0.3
Analyzed nutrient composition				
DM (%)	61.9	61.8	61.7	61.9
Organic matter (% of DM)	90.5	90.2	90.0	89.6
Crude protein (% of DM)	14.3	14.2	14.1	14.1
NDF (% of DM)	50.1	50.5	51.0	51.4
ADF (% of DM)	21.9	22.6	22.5	22.9
Ether extract (% of DM)	1.53	1.49	1.57	1.59
Water-soluble carbohydrates (% of DM)	6.39	7.30	8.05	8.74
Calcium (% of DM)	0.61	0.62	0.63	0.63
Phosphorus (% of DM)	0.45	0.45	0.45	0.45
Sulfur (% of DM)	0.21	0.24	0.26	0.29
NFC (% of DM)	24.2	24.0	23.6	22.6
TDN2) (% of DM)	67.8	67.4	67.8	67.8
Dietary pH	4.92	4.88	4.80	4.74

DM, dry matter; FVD, fruit and vegetable discards, mixed with 6 g sodium metabisulfite/kg wet FVD and aerobically challenged for 7 d; TMR, total mixed ration; NDF, neutral detergent fiber; ADF, acid detergent fiber; NFC, non-fibrous carbohydrates; TDN, total digestible nutrients.

1) Per kg contained: vitamin A, 5,000,000 IU; vitamin D, 500,000 IU; Zn, 8 g; Mn, 6.5 g; Fe, 3.3 g; Cu, 1.2 g.

2) The TDN content of FVD was estimated using the equation of Conrad et al [12], considering crude protein, ether extract, ash, NDF, and lignin contents of FVD.

**Table 4:** Gas production and ruminal metabolites of diets with different levels of FVD

Items	Level of FVD in TMR (% of DM)				SEM	p-value		
	
	0	1.9	3.8	5.7		Treatment	Linear	Quadratic
Gas at t_1/2_ (mL)	101	94.1	94.6	95.3	1.22	0.03	<0.01	0.12
ITOMD (mg)	321	317	315	320	3.4	0.33	0.77	0.19
Ruminal pH	6.53	6.51	6.53	6.50	0.031	0.31	0.37	0.29
NH_3_-N (mM)	8.71	8.44	8.75	8.61	1.392	0.77	0.67	0.73
Lactate (mM)	1.93	2.02	1.95	1.97	0.093	0.45	0.62	0.33
Partitioning factor1) (mg/mL)	3.18	3.37	3.32	3.35	0.062	0.07	0.12	0.09

FVD, fruit and vegetable discards, mixed with 6 g sodium metabisulfite/kg wet FVD and aerobically challenged for 7 d; TMR, total mixed ration; DM, dry matter; SEM, standard error of the mean; ITOMD, *in vitro* true organic matter degradability.

1) Calculated as the ratio of mg true digested organic matter to mL GP*t*_1/2_.

**Table 5:** Apparent nutrient digestibility, nitrogen metabolism, and voluntary feed intake of sheep fed diets with different levels of FVD

Items	Level of FVD in TMR (% of DM)				SEM	p-value		
	
	0	1.9	3.8	5.7		Treatment	Linear	Quadratic
Apparent digestibility (%)								
Organic matter	70.6	71.6	71.3	72.1	0.98	0.45	0.33	0.37
Crude protein	78.9	80.2	79.9	79.8	1.01	0.25	0.13	0.71
Ether extract	78.2	80.1	79.2	80.0	2.11	0.46	0.53	0.91
Neutral detergent fiber	63.1	64.7	64.3	65.6	1.32	0.19	0.06	0.79
Acid detergent fiber	49.1	48.9	49.5	50.7	1.11	0.21	0.33	0.76
Nitrogen (N) metabolism								
Intake (g/d)	23.0	22.8	22.7	22.7	0.53	0.54	0.51	0.92
Fecal (g/d)	6.99	6.97	7.04	7.15	0.331	0.71	0.42	0.56
Urinary (g/d)	8.71	7.98	8.20	8.45	0.661	0.43	0.38	0.11
Retained N (g/d)	7.29	7.85	7.44	7.08	0.420	0.33	0.27	0.20
Fecal N/N intake (%)	30.4	30.5	31.1	31.5	0.92	0.53	0.50	0.14
Urinary N/N intake (%)	37.9	34.9	36.2	37.3	1.77	0.32	0.44	0.21
Retained N/N intake (%)	31.7	34.5	32.8	31.2	1.65	0.27	0.20	0.44
Voluntary feed intake (kg DM/d)	1.56	1.44	1.42	1.47	0.074	0.13	0.42	0.18
Voluntary feed intake (g DM/kg BW^0.75^/d)	84.2	77.6	76.8	81.4	4.14	0.35	0.49	0.20
Voluntary water intake (kg/kg DMI)	1.92	1.87	2.19	2.15	0.202	0.17	0.90	0.71
Fecal score1)	1.62	1.48	1.57	1.44	0.151	0.61	0.33	0.88

FVD, fruit and vegetable discards, mixed with 6 g sodium metabisulfite/kg wet FVD and aerobically challenged for 7 d; TMR, total mixed ration; DM, dry matter; SEM, standard error of the mean; DMI, dry matter intake.

1) Fecal consistency was determined individually using a five-point scale, where 1 = firm, dry, well-formed pellet and 5 = diarrhea.

**Table 6:** Blood metabolites of sheep fed diets with different levels of FVD

Items	Level of FVD in TMR (% of DM)				SEM	p-value		
	
	0	1.9	3.8	5.7		Treatment	Linear	Quadratic
Total protein (g/dL)	6.58	6.18	6.73	6.55	0.289	0.56	0.71	0.69
Globulin (g/dL)	3.43	3.10	3.53	3.33	0.232	0.65	0.91	0.80
Albumin (g/dL)	3.15	3.08	3.20	3.23	0.081	0.73	0.46	0.63
Creatinine (mg/dL)	0.72	0.76	0.72	0.74	0.026	0.44	0.87	0.81
Urea (mg/dL)	41.5	39.8	40.1	41.9	1.55	0.38	0.36	0.15
Glucose (mg/dL)	71.5	71.8	71.3	70.6	1.51	0.62	0.23	0.63
Ceruloplasmin (mg/dL)	8.27	7.82	8.17	7.93	0.362	0.31	0.18	0.48
Phosphorus (mg/dL)	5.18	5.20	5.08	5.10	0.123	0.79	0.39	0.68
Potassium (mmol/L)	4.78	4.90	4.83	4.98	0.11	0.50	0.25	0.90
Sodium (mmol/L)	147	149	148	148	1.4	0.29	0.93	0.12
Chlorine (mmol/L)	107	108	108	105	1.8	0.34	0.33	0.15
Lactate dehydrogenase (U/L)	559	605	583	597	30.4	0.59	0.53	0.56
Alkaline phosphatase (U/L)	228	251	232	247	20.1	0.14	0.58	0.93
Alanine aminotransferase (U/L)	10.8	11.2	11.5	11.8	0.56	0.15	0.25	0.70
Aspartate aminotransferase (U/L)	105	107	111	110	6.1	0.75	0.43	0.72
Vitamin A (μmol/L)	1.30	1.27	1.28	1.31	0.086	0.37	0.41	0.23
Vitamin E (μmol/L)	0.86	0.93	0.89	0.91	0.063	0.51	0.53	0.61
TAC (μmol Trolox equivalent/mL)	1.63	1.70	1.67	1.64	0.038	0.16	0.29	0.23

FVD, fruit and vegetable discards, mixed with 6 g sodium metabisulfite/kg wet FVD and aerobically challenged for 7 d; TMR, total mixed ration; DM, dry matter; SEM, standard error of the mean; TAC, total antioxidant capacity.
